# Development of Non-Targeted Mass Spectrometry Method for Distinguishing Spelt and Wheat

**DOI:** 10.3390/foods12010141

**Published:** 2022-12-27

**Authors:** Kapil Nichani, Steffen Uhlig, Bertrand Colson, Karina Hettwer, Kirsten Simon, Josephine Bönick, Carsten Uhlig, Sabine Kemmlein, Manfred Stoyke, Petra Gowik, Gerd Huschek, Harshadrai M. Rawel

**Affiliations:** 1 QuoData GmbH, Prellerstr. 14, D-01309 Dresden, Germany; 2 Institute of Nutritional Science, University of Potsdam, Arthur-Scheunert-Allee 114-116, D-14558 Nuthetal, Germany; 3 QuoData GmbH, Fabeckstr. 43, D-14195 Berlin, Germany; 4 Bundesinstitut für Risikobewertung, Max-Dohrn-Str. 8-10, D-10589 Berlin, Germany; 5 Akees GmbH, Ansbacher Str. 11, D-10787 Berlin, Germany; 6 Bundesamt für Verbraucherschutz und Lebensmittelsicherheit, Diedersdorfer Weg. 1, D-12277 Berlin, Germany; 7 IGV-Institut für Getreideverarbeitung GmbH, Arthur-Scheunert-Allee 40/41, D-14558 Nuthetal, Germany

**Keywords:** non-targeted methods, LC-MS, fingerprinting, machine learning, convolutional neural networks, wheat, spelt, food fraud

## Abstract

Food fraud, even when not in the news, is ubiquitous and demands the development of innovative strategies to combat it. A new non-targeted method (NTM) for distinguishing spelt and wheat is described, which aids in food fraud detection and authenticity testing. A highly resolved fingerprint in the form of spectra is obtained for several cultivars of spelt and wheat using liquid chromatography coupled high-resolution mass spectrometry (LC-HRMS). Convolutional neural network (CNN) models are built using a nested cross validation (NCV) approach by appropriately training them using a calibration set comprising duplicate measurements of eleven cultivars of wheat and spelt, each. The results reveal that the CNNs automatically learn patterns and representations to best discriminate tested samples into spelt or wheat. This is further investigated using an external validation set comprising artificially mixed spectra, samples for processed goods (spelt bread and flour), eleven untypical spelt, and six old wheat cultivars. These cultivars were not part of model building. We introduce a metric called the D score to quantitatively evaluate and compare the classification decisions. Our results demonstrate that NTMs based on NCV and CNNs trained using appropriately chosen spectral data can be reliable enough to be used on a wider range of cultivars and their mixes.

## 1. Introduction

Public awareness around food fraud and food authenticity is mainly driven by high-visibility media discussions, e.g., in connection with public health consequences or when a large-scale operation is uncovered and the ensuing scandal brings disrepute to companies or regulatory authorities [1,2]. However, even when not topical, food fraud is widespread and exacts considerable economic costs [3,4]. Its manifold manifestations include adulteration, mislabeling, dilution, substitution, etc. [5]. Establishing procedures and quality indicators to detect food fraud, therefore, continues to be an important and urgent task [4].

Being one of the most important food crops in the world, wheat, its varieties, and derived products are defenseless against rampant fraud [6]. Analytical testing for determination of authenticity and detection of fraud is an important control measure to identify, monitor, and act—to ensure consumer safety and punish the perpetrators [7]. The testing can range from differentiating grain types, e.g., durum, einkorn, spelt, etc. [8], tracing geographic identity [9], especially protected geographic identity, e.g., that of Fränkischer Grünkern (a spelt product) [10], testing the presence of adulterants [11,12], and checking crop growing or harvesting conditions (e.g., organic wheat) [13], among others.

It is reported that spelt (*Triticum spelta*) is one of the three ancient wheats that are considered to be the ancestors of modern wheat. The other two are emmer and einkorn. Genetic data suggests that spelt can occur from the hybridization of bread wheat and emmer wheat, but only after the first *Aegilops-tetraploid* wheat hybridization. The considerably later development of spelt in Europe might be attributed to a later, second hybridization between emmer and bread wheat [14]. Hence, for centuries, spelt (or “*Dinkel*” in German) has remained a major grain in the DACH region (Germany, Switzerland, and Austria) [15]. They are very resilient to austere irrigation conditions while having favorable digestive and nutritional values [16]. As a consequence, its demand and market price are on the rise. Lately, spelt has become part of many bakery products, pasta, noodles, and even beer [17]. In light of accelerating demand and consumption of spelt and spelt-derived products, it is hard to ignore the possibility of market-driven fraudulent practices. As these grains command a premium price, there is an economic benefit to devising new tactics for adulteration, tampering, substitution, etc. Thus, there is a need to address this through the development of new methods for distinguishing spelt and wheat [18,19]. At this point, it is necessary to mention that addressing the economic or nutritional benefits of spelt over wheat is outside the scope of this work.

Spelt is mostly referred to by its phylogenetic and morphological characteristics, but in practice, unequivocal identification of spelt based on physiological properties is non-trivial [20,21,22,23]. Perhaps this is because of its close botanical relationship with wheat and crossbreeding over hundreds of years. Consequently, determining whether a cultivar can be classified as spelt is challenging [22]. Switzerland maintains guidelines laid out through IP-SUISSE and Bio-Suisse in cooperation with IG Dinkel to regulate the growing and selling of certain old spelt species (Urdinkel in German) [24]. Thus, the questions arise: which cultivars are true spelt, and how can they be determined?—the latter being the more challenging question. The general European Union (EU) legal framework, as put forward in regulations such as 2017/625 and 1169/2011, aims to ensure food safety and consumer protection by compelling producers to correctly label ingredients and their sources [25,26]. In this case, product labeling must be combined with an authentication analysis of grain ingredients and additives. Under the circumstances of the lack of consensus on which cultivars are truly spelt, the challenge of performing an authentication analysis is formidable. The challenges of discerning species only snowball when it comes to processed goods, such as bakery items. In Germany, there is a guideline (Leitsätze des Deutschen Lebensmittelbuchs für Brot und Kleingebäck) that serves as a guiding principle for the manufacture and sale of spelt bread [27]. It states that spelt bread must contain at least 90% spelt. Thereby, processed goods will certainly contain wheat along with spelt, which only further complicates the process of identifying or detecting spelt for authenticity testing. Adding newer cultivars of spelt to the mix, such as “pre-spelt,” or “wheat-spelt” crossed cultivars (together referred to in this work as “untypical spelts”), only increases the challenge to unequivocally define what is spelt and what is not.

Non-targeted methods (NTMs) are being increasingly developed and deployed in the detection of food fraud and ratifying the authenticity of food substances [28,29,30]. An NTM encompasses analytical measurement, resulting in, e.g., a highly resolved fingerprint (referred to herein as the wet lab procedure), followed by mathematical modeling and data evaluation (referred to as the dry lab procedure), without laying a special spotlight on predetermined analytes of interest [31].

In the wet lab part, mass spectrometry (MS) based testing is a dominant and useful kind of NTM [32]. Coupling with liquid chromatographic (LC) separation and connection to a high-resolution (HR) mass analyzer like the time of flight (TOF) enables precise mass determination at different retention times (Rt) [33]. The resulting LC-HRMS spectra are useful to capture the slightest differences between sample populations, which arise because peptides and proteins in food substances are expressed differentially, not only due to inherent genetic composition but also due to external factors that might have their genesis in nature (such as soil type and quality, climatic conditions) or be caused by humans (agricultural practices, adulteration, mixing, etc.) [34].

The other important component of an NTM is the dry lab, which includes statistical modeling [31]. Given the complexity and size of the measurement data that is generated with LC-HRMS, there is a need to resort to contemporary machine learning methods like neural networks [35,36]. Neural networks have become increasingly popular in different application areas, including MS, because several studies have been reported in the literature exploiting neural networks for MS data. The strategies in the reported studies can be essentially grouped by the different tasks undertaken, for instance, (1) peak pre-processing such as normalization [37] and peak alignment [38], (2) evaluation of peak features [39,40,41,42], (3) spectra prediction [43], (4) spectral annotation and molecular structure prediction [44,45], and (5) classification of samples based on the associated spectra. The fifth strategy can be divided into two types: one that utilizes a peak list or feature list, and the other that uses the entire spectrum. With the latter, a few reports have explored using 1-d MS spectra with convolutional neural networks (CNN) [46,47,48].

**Figure 1:**
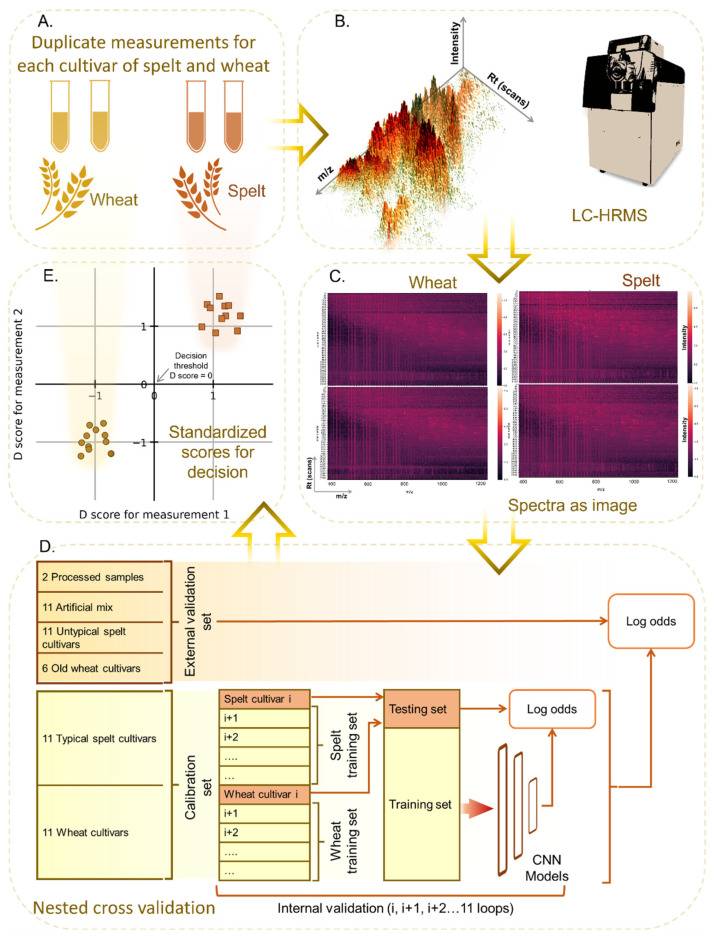
Schematic illustration showing the high-resolution liquid chromatography mass spectrometry (LC-HRMS) based non-targeted method (NTM) proposed and developed in this work to distinguish spelt and wheat. (**A**) Duplicate samples for each cultivar of spelt and wheat were prepared, and (**B**) measured using a SCIEX ESI-TripleTOF 5600 with SWATH acquisition. (**C**) The 2-D spectra are depicted as an image with mass by charge (*m*/*z*) as the x-axis, retention time (Rt) as the y-axis, and intensity as the z-axis. The exemplary images shown are the duplicate measurement spectra for Bernstein wheat and Badekrone spelt. (**D**) A nested cross validation (NCV) approach was adopted with a separate calibration and external validation set. Convolutional neural network (CNN) models trained with 11-fold internal validation. The log odds values are calculated using the output probabilities of the CNN models. (**E**) Using the log odds, a standardized value called the D score is calculated and plotted on a Youden plot. The scores help in the identification of the tested sample. A decision threshold score of zero is used in this case. The plot shows exemplary point clouds for the spelt (orange squares) and wheat (brown circles) cultivars.

CNNs are a type of neural network that have been shown to be powerful for image processing tasks like face classification and recognition [49,50]. Herein, we aim to apply these capabilities to parse HR mass spectra with normalized mass windows (SWATH acquisition) [51] and, thereby, classify spelt or wheat (as illustrated in Figure 1A). An image can be formed from the 2-d spectral data using the peak height intensities for each mass/charge (*m*/*z*) and Rt (see Figure 1B,C). The combination of 2-D spectral data with CNNs as an NTM for the classification of spelt and wheat has not been previously reported, to the best of our knowledge. To this end, in this work, we describe an NTM in which the wet lab component captures the food fingerprint (peptide marker profile) using LC-HRMS and the dry lab component uses CNN to learn the differences between the fingerprints and eventually classify the tested sample. The predicted outcomes are compared using a new metric that we call the D score.

## 2. Materials and Methods

### 2.1. Description of Spelt and Wheat Samples

Samples for all spelt and wheat cultivars were kindly sourced and provided by the Institut für Getreideverarbeitung (IGV) GmbH, Nuthetal, Germany. Eleven cultivars each of typical spelt and wheat were used to train the CNN models. The distinction of whether it is spelt or wheat was made according to investigations of their marker peptide profiles, as previously described elsewhere [52]. For the list of eleven cultivars each for spelt and wheat, see supplementary Table S1. Each of the cultivars was measured in duplicate on different days (different runs). Together, 44 MS1 spectra constitute the “calibration dataset,” i.e., all the spectra that were used to train the CNN models. In this communication, we choose to refer to this as the calibration dataset in accordance with other reports [46,53]. For each of the internal validation folds, the calibration set was split into the training and testing sets (see Figure 1D). The term “training of models” refers to obtaining the weights and biases of the neural network through a process of back propagation [54]. Further details are described in Section 2.3.

Two processed samples were prepared to keep in mind commonly available processed spelt goods. The first sample was a mixture of spelt flour made of *Oberkulmer Rotkorn* with 10% wheat flour T405. The second sample was a spelt bread baked using spelt flour T630 with 10% soft wheat flour T550. To simulate the flour and bread samples, an artificial spectral mix was generated by the weighted addition of two spectra. Duplicate measurements for each of the eleven wheat cultivars were 10% down-weighted and added to 90% of the spectral intensities of one spectrum of *Oberkulmer Rotkorn* spelt to yield eleven pairs of artificial mix spectra. As per the guiding principle for the manufacture and sale of spelt bread [27], which states that the spelt bread must contain at least 90% spelt, the maximum possible wheat content of 10% was chosen.

Additionally, eleven cultivars of untypical spelt were sourced. These cultivars of spelt are known to be either “newer” cultivars of spelt or wheat-spelt crosses; hence, they are collectively referred to herein as “untypical spelt.” Furthermore, six wheat cultivars were also sourced whose pedigrees can be be traced to the late 18th to early 19th centuries, hence being referred to herein as “old wheat” cultivars. For a list of untypical spelt and old wheat cultivars, see supplementary Table S1. Together, these constitute the “external validation dataset,” which consists of unseen data used to test the trained models. Just like the calibration set, for each of the mixture samples and cultivars, duplicate measurements were performed.

### 2.2. Wet Lab Procedure

This section briefly describes the sample preparation and LC-HRMS measurements as part of the wet lab procedure. The detailed MS procedure has been reported as part of previously conducted targeted studies [51,52].

#### 2.2.1. Sample Preparation, Protein Digestion and Purification

All buffer solutions and dilutions were prepared with water suitable for LC-MS analysis. Each sample was weighed to 1.0 ± 0.001 g in a 50 mL centrifuge tube, to which 10 mL of extraction buffer was added. Extraction buffer was prepared with 100 mM ammonium bicarbonate, 4 M urea, and 5 mM 1,4-Dithiothreitol (DTT) (all from Carl Roth GmbH, Karlsruhe, Germany). The tube was shaken at room temperature for 1 h using an overhead shaker, after which it was centrifuged at 4000 *g* for 5 min. 2 mL of the supernatant was transferred to a 15 mL centrifuge tube and centrifuged again at 7000× *g* for 5 min. 1 mL of the supernatant was removed and transferred to another 15 mL centrifuge tube, to which 30 µL of 0.5 M Iodoacetamide (IAA) solution was added. 0.5 M IAA solution was prepared fresh, as it is light sensitive, by dissolving 11.55 mg of IAA (Sigma-Aldrich, Taufkirchen, Germany) in 1.25 mL water. The resulting solution was incubated for 20 min by shaking at 50 °C, after which (a) 3000 µL of digestion buffer and (b) 100 µL of chymotrypsin solution (from bovine pancreas for enzymatic digestion purchased from Sigma Aldrich, Taufkirchen, Germany) were added. This is followed by incubation of the reaction mixture overnight at 25 °C. The (a) digestion buffer was prepared by dissolving 1.304 g ammonium bicarbonate in 25 mL Acetonitrile (ACN) (both from Carl Roth GmbH, Karlsruhe, Germany) and diluting with 140 mL of water. The (b) chymotrypsin solution was freshly prepared using activated chymotrypsin (>1000 USP-U/mg) (Carl Roth GmbH, Karlsruhe, Germany) at a concentration of 8 mg /mL. The digestion reaction was stopped by adding 100 µL of 40% formic acid (FA) (Carl Roth GmbH, Karlsruhe, Germany). The extract obtained was stored for at least 1 h in the freezer at −20 °C, so that most of the fat or wax components precipitated. The reaction tubes were then centrifuged at 7000× *g* for 2 min.

The sample extract was desalted and concentrated using an SPE column (Carl Roth GmbH, Karlsruhe, Germany). For this purpose, the SPE columns were conditioned with 6 mL of buffer A followed by 6 mL water. Buffer A was made by mixing 100 mL water, 100 mL can, and 200 µL FA. Then the entire sample extract was added to the column and unbound components were washed out by subsequent rinsing with 6 mL of buffer B. Buffer B was prepared by mixing 200 µL water with 200 µL FA. The eluted peptides were then concentrated to dryness under nitrogen at 30 °C and resuspended in a mixture of 450 µL buffer B and 50 µL buffer A. Lastly, the mix was centrifuged for 2 min at 7000× *g*. The supernatant was diluted with buffer B in a ratio of 1:100 and then measured.

#### 2.2.2. Liquid Chromatography Mass Spectrometry (LC-MS)

Data were acquired using ultra-high performance liquid chromatography triple time of flight mass spectrometry (UHPLC Triple ToF) (MS/MS) consisting of a micro-flow UHPLC expert microLC 200 with an autosampler CTC Pal system and a SCIEX electrospray ionization (ESI) TripleTOF 5600 with SWATH (sequential window acquisition of all theoretical fragment-ion spectra) acquisition. HRMS data acquisition of MS/MS data was done using data-independent acquisition (DIA-SWATH) [55]. Although MS2 SWATH data was also acquired, it was not utilized for the analysis shown in this work. As mentioned earlier, every measurement was performed in duplicate.

### 2.3. Dry Lab Pipeline

#### 2.3.1. Spectral Data Preparation

The acquired data were first converted to the mzXML file format from the WIFF and WIFFSCAN formats using ProteoWizard [56]. All MS datasets were used without undergoing any preprocessing (e.g., peak alignment, baseline correction) or feature selection steps. The mzXML file was read in the Python programming language (python.org), and the MS1 spectra were aggregated to integer mass accuracy. The resulting data were a matrix of size 1375 (number of scans) and 801 (values of *m*/*z* ranging from 400 to 1200 Da). The aggregation of spectra was performed to make it manageable for CNN model training on a personal computer. The data matrices were obtained for all the samples in the calibration set and external validation set, which were then used as input to the CNN models. Each scan was z-normalized, i.e., subtract the mean of a scan from every peak intensity value and divide by the standard deviation (SD) of the scan.

#### 2.3.2. Nested Cross Validation (NCV)

Central to the analysis pipeline was the NCV approach shown in Figure 1D. The calibration set comprised eleven cultivars each for typical spelt and wheat as the two classes for the CNN model classifier. In this, separate models were trained with a training set comprising duplicate spectra for (randomly chosen) ten cultivars each of typical spelt and wheat (totaling forty spectra) and tested on the spectra for the remaining eleventh cultivar for typical spelt and wheat (totaling four spectra). For instance, in the first fold, spectra for *Badekrone* spelt and *Bernstein* wheat cultivars were kept aside for testing the model trained on the remaining spectra of the cultivars. In the next fold, spectra for *Badensonne* spelt and *Brilliant* wheat cultivars were kept aside for testing the model trained on the spectra for the remaining cultivars. In this way, eleven models were trained, corresponding to each fold of the internal validation loop. In other words, every cultivar in the calibration set was used once to test the trained models. The NCV procedure is advantageous because it can deal with the availability of a limited number of distinct samples (cultivars), each having a large number of features (peaks). For the external validation dataset, every spectrum was run through models for each fold of the NCV to obtain a classification outcome in the form of a probability. The final classification probability for the external validation spectra was obtained by averaging across all the model outcomes (i.e., the average of eleven models’ outcomes).

#### 2.3.3. Neural Network Analysis

In this communication, a short description is provided for how the neural network was constructed, assuming that the reader is aware of terms used in the field. The reader is referred to rich literature available elsewhere for (a) the theoretical fundamentals behind neural networks and (b) an exhaustive review on the types of neural network architectures [57,58,59,60,61]. A shallow CNN architecture was used with convolutional layers and pooling layers, each of which was setup using standard settings [62]. All programming was done in Python (python.org) using the Keras and Tensorflow libraries [63,64]. Four convolution layers were stacked together to hierarchically capture the inherent patterns within the spectra. The convolution layers were interspersed with “maximum pooling” layers, which help reduce the effect of spectral noise in the learned features and emphasize the larger peak intensities [65]. Together, the above-described apparatus tries to automatically extract the “features”—which, in this context, are the spectral peaks (or their combinations). We hypothesize that the features learned by the CNNs directly help to identify a particular class (spelt or wheat), which otherwise would have been done by a human expert.

For each fold of internal validation, the calibration set was split into a training and a testing set. According to the NCV approach, CNN models were trained on the training set and then checked using the testing set. The CNN was trained using gradient descent, which minimizes a loss function by calculating its partial derivative with respect to the learnable parameters through backpropagation and iteratively updating them until they converge for each layer [46,47,54]. The output of the CNN was a probability value (used for the D score calculation as described in the next section), based on which a binary classification was obtained (spelt or wheat). The performance of the classifier was tracked by looking at the confusion matrix, i.e., counts of true positives (TP), true negatives (TN), false negatives (FN), and false positives (FP). Using these values, Matthew’s correlation coefficient (MCC) was calculated according to Equation (1). MCC = 1 means a perfect prediction, whereas MCC = −1 means completely flipped (incorrect) predictions.

(1) $$MCC=\frac{TP\times TN-FP\times FN}{\sqrt{\left(TP+FP\right)\left(TP+FN\right)\left(TN+FP\right)\left(TN+FN\right)}}$$

In this study, the features available to train the CNNs were ample, i.e., ~1 million per measurement, while the number of cultivars per class was limited (11 each). Hence, it was important to keep the models “simple” and avoid extensive hyperparameter tuning. Hyperparameters can be thought of as knobs and dials available to design CNNs and determine how they are trained. For instance, the number of layers in a CNN, the learning rate of the gradient descent algorithm, the number of epochs, etc. [40,57]. Tuning these parameters can result in model predictions being overly dependent on the underlying training data, i.e., lead to overfitting. This means that when models are trained for a set of cultivars, they may not perform very well on other types of cultivars.

### 2.4. Decision Based on D Scores

The newly proposed quantitative score, called the D score, is a measure of the classification outcome that can be easily compared for different types of samples, experimental runs, models, or even laboratories. The classification outcome from the CNN models was extracted in the form of probabilities $\left(p_{i}\right)$. The probabilities were converted to log odds ratios. A linear transformation was then performed on the log odds ratio values to scale the values such that the mean values of the spelt and wheat classes are +1 and −1, respectively (Equations (2)–(4)). The resultant values are referred to as “D scores.” The linear transformation parameters (λ, θ) were obtained based on the calibration set of samples, i.e., using the means of log odds for spelt (${\bar{\mu }}_{spelt}$) and wheat (${\bar{\mu }}_{wheat}$). The calculated D scores for the duplicate measurements were then plotted on a Youden plot, as shown in Figure 1E. A Youden plot is essentially a scatter plot that helps to visualize and analyze data when two measurement runs on the same type of sample (in this case, the cultivar).

(2) $$D_{i}=ln\left(\frac{p_{i}}{1-p_{i}}\right)\times \lambda +\theta ;for\;i^{th}\;measured\;sample$$

(3) $$where,\;\lambda =\frac{2}{{\bar{\mu }}_{spelt}-{\bar{\mu }}_{wheat}}\;and$$

(4) $$\theta =1-\lambda \times {\bar{\mu }}_{spelt}$$

The decision for classification would be based on a decision threshold, which is chosen to be zero in this study. Hence, when the D score is positive ($D_{i}>0)$, then spelt, and when it is negative ($D_{i}<0)$, then wheat. In comparison to a qualitative binary classification (yes/no) outcome, D scores offer three main advantages. First, the distribution of D scores allows one to evaluate the performance of the model or the method as a whole by calculating the variation of the scores within a class. This is further discussed in Section 3.3. Secondly, it allows direct comparison of samples and informs about the relationship between the compared samples. For instance, 2 samples with D scores of 0.8 and 1 are expected to be closely related (from their prediction classification) compared to samples with D scores of +0.8 and −0.8. This is further illustrated in Section 3.2. Finally, D scores are model- and class-agnostic. Hence, the procedure for calculation and interpretation of D scores will not change on (a) replacing the neural network model with another (type of) classifier and (b) when the classes are changed from spelt or wheat to any other generic class A or B (for example, a white wine from Germany and a white wine from France).

## 3. Results

### 3.1. Wet Lab LC-HRMS Measurements

With the purpose of utilizing complete and raw spectra from the LC-MS measurements, the 2-D spectra for each sample were obtained. The 2-D spectrum can be visualized as an image. Figure 1C shows exemplary heatmap images for duplicate measurements of spelt and wheat. The x-axis of the image shows the *m*/*z* and the y-axis shows the scans corresponding to different retention times, and the intensity of the values is indicated by the color map. The heatmaps are plotted with power-law normalization of the intensity for better visual contrast. Even on closer inspection, distinction between the patterns (or fingerprints) is hard to make only with the human eye. Hence, the need for devising suitable models that are able to parse the data, capture the underlying patterns, and help distinguish the food items (here, spelt and wheat) is apparent. These images were used as input for the dry lab model.

### 3.2. Internal Validation: Youden Plot with the D Scores for Calibration Set

**Figure 2:**
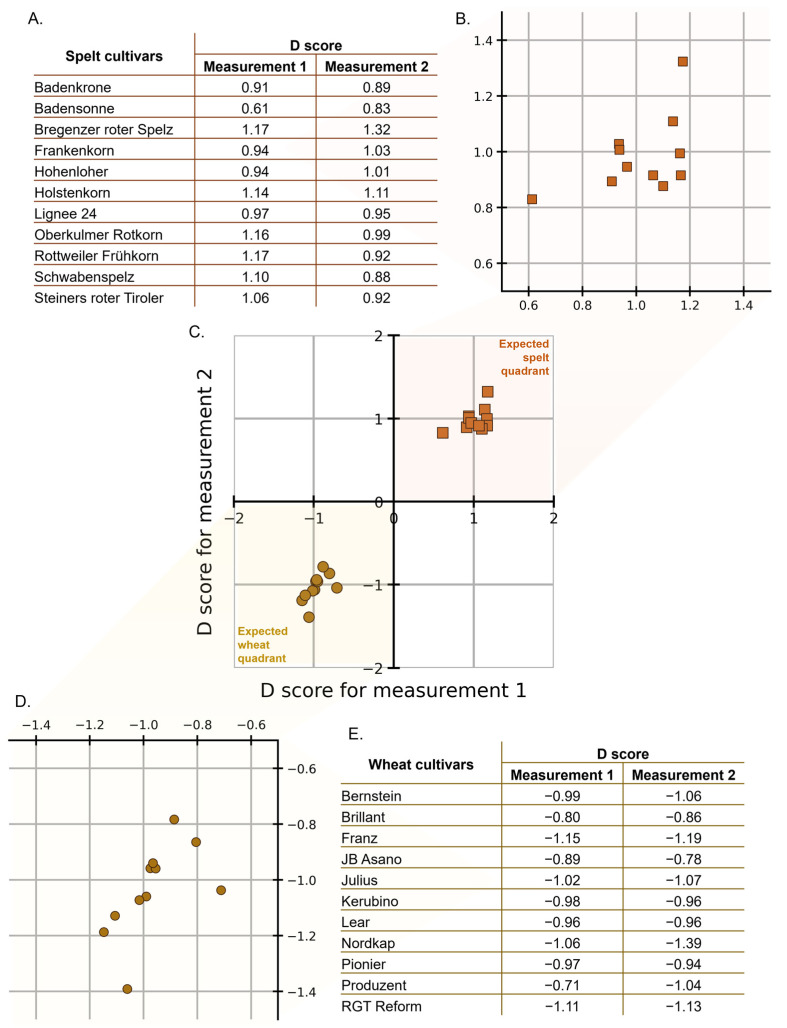
The D scores for the spelt cultivars (orange squares) and for wheat cultivars (brown circles) in the calibration set are plotted. (**A**) List of spelt cultivars along with their D scores. (**B**) A magnified view of the Youden plot for spelt cultivars. (**C**) Youden plot with the D scores. (**D**) A magnified view of the Youden plot for wheat cultivars. (**E**) List of wheat cultivars along with their D scores.

After going through the NCV procedure for internal validation, D scores were obtained for each of the spectra in the calibration set. Recall, two extracted samples were measured, hence, two sets of spectra are available for each cultivar, and each cultivar is tested once with a model trained on cultivars other than itself. Hence, this gives us a D score for the entire calibration set. The λ and θ values calculated according to Equations (2) and (3) are −0.13 and −0.02, respectively (see supplementary results Section S2.1). Figure 2A shows a list of spelt cultivars, where each cultivar is indicated by a point in the magnified cluster of the plot shown in Figure 2B. Figure 2C shows a Youden plot with point clouds for the spelt (orange squares) and wheat (brown circles) cultivars in the calibration set. Figure 2D shows a magnified cluster of points where each point on the plot represents a wheat cultivar that is listed in Figure 2E. The Youden plot allows us to intuitively establish the extent of discrimination (a) between the samples of the two classes (spelt and wheat) and (b) among the samples of the same class.

The lack of any overlap between the point clouds directly shows the high discriminatory power of the trained models. Considering zero as the decision threshold for the D scores, when a D score is positive for both measurements, it lies in the first quadrant (top right) and is predicted to be spelt. Likewise, when it is negative for both D scores, it lies in the third quadrant (lower left) and is predicted to be wheat. Here, the advantage of the D score is evident in being able to immediately identify if the classification outcome is spelt or wheat (for a list of D scores for the calibration set, see Supplementary Table S2A,B). If visual proof is insufficient, the classification performance can be summarized using the Matthews correlation coefficient (MCC), which is suggested to be the most informative of all the different classification metrics [66]. MCC of +1 is obtained, which shows complete agreement between the true and predicted classes, making the high classification performance very evident. The separation in the D score point clouds shows that CNNs prove effective in learning visual representations of 2-D spectral data that are passed as images. It is expected that convolution layers are able to capture the local shifts in the peaks (that are typically then aligned, corrected, etc. in spectral preprocessing).

### 3.3. Precision Parameters

It is essential to ensure that the discriminatory power remains adequate (a) when applied to other sets of data than the training set, covering the entire population falling under the scope of the method, and (b) under all in-house testing conditions or when applied to data from different laboratories. Using the D scores, various precision estimates can be obtained based on concepts laid out in ISO 5725-3 [67]. Note that the standard describes precision parameters that are given for a sample, but in this context the parameters are provided for the class (e.g., spelt or wheat and not for a specific cultivar). Here we calculate the classification SD (the variation of D scores for cultivars within a class) and intermediate SD (the average variation of D scores for several measurements (at least 2) of the same cultivar under intermediate conditions, averaged across cultivars of the same class). The precision estimates for the D score can be obtained by using the approach described in previous reports [68,69] (see supplementary Table S3).

The single laboratory classification SD is used to check whether the decision threshold can be considered reliable for the whole population falling within the scope of the classification method. SD values of 0.393 and 0.391 are obtained for spelt and wheat, respectively. If we assume that D scores are normally distributed within each of the two classes, then with a mean value of 1 and SD of 0.393, the risk of misclassification for spelt, i.e., a value below zero, would have a probability of $\Phi \left(\frac{-1}{0.393}\right)\approx 0.5\%$. Similarly, the risk of misclassification for wheat, would be $1-\Phi \left(\frac{1}{0.391}\right)\approx 0.5\%$. Here, Φ denotes the cumulative distribution function of the standard normal distribution. There is no indication that the point clouds of D scores for each class are not normally distributed. Thus, the risk of misclassification is very low (<1%).

With the intermediate SD, the in-house reproducibility of the D score can be described. We obtained an intermediate SD of 0.075 and 0.074 for spelt and wheat, respectively, which means that the analytical variability is almost equal to the variability between different cultivars. It can, therefore, be stated that the analytical variability is more than sufficient for the purpose of classification between wheat and spelt; on the other hand, the differences within the spelt cultivars studied are very small and cannot be precisely measured with the D score. The next section describes how the trained models perform on external validation samples. Predictions on external validation samples were performed using all the models trained in the internal validation NCV loops.

### 3.4. External Validation Set: Processed Goods and Artificial Mixes

**Figure 3:**
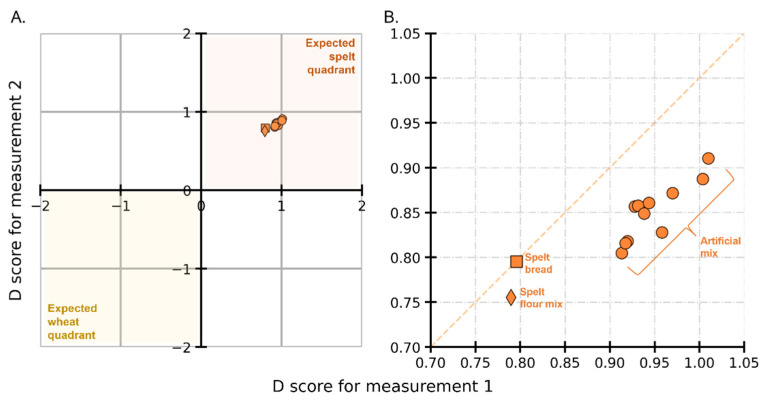
Youden plot showing the D scores for processed goods and artificial mix in the external validation set in (**A**) and a magnified section in (**B**). Spelt bread (orange square), spelt flour mix (orange diamonds), and artificial spectral mix (orange circles) are shown to be correctly predicted as spelt.

Even with the limited number of distinct cultivars used for training a CNN model, the present study was designed to determine whether successful classification models can be built using LC-HRMS spectra, and thereby laying the groundwork for an NTM that can be used in routine (e.g., for official control). The models trained with typical spelt and wheat varieties are put to the test by using real-world processed goods. Remember that each of the eleven internal validation models provided an output prediction, which was then averaged to get an average D score for each external validation sample. Figure 3A shows spelt bread (orange square) and spelt flour mix (orange diamond) in the expected spelt quadrant, hence showing the correct classification. Figure 3B shows a magnified view of the points (for a list of D scores, see Supplementary Table S4). The resulting D scores for both measurements of spelt bread are around 0.79 and the scores for the duplicate measurements of spelt flour mix are around 0.78 and 0.75. Together, the D scores for processed goods indicate a correct prediction.

Turning now to predicting the artificially generated spectral mixes, Figure 3 shows the D scores (orange circles) for each of the eleven wheat cultivars whose spectra were 10% downweighed and added to 90% of the spectral intensities of *Oberkulmer Rotkorn* spelt. The average D score for these eleven points is around 0.9. Interestingly, the point cloud for the artificial mix is further away (top right) from the actual processed goods. In other words, the predictions from CNN models are relatively (and marginally) more confident about the artificial mix being spelt than the spelt bread and flour. Perhaps this is because the spectra for bread and flour have a more complex fingerprint than the one resulting from the linear combination of their constituents. In summary, the predictions on the external validation set show that successful distinction can be made even on processed spelt samples.

### 3.5. External Valdiation with Untypical Spelt Cultivars

**Figure 4:**
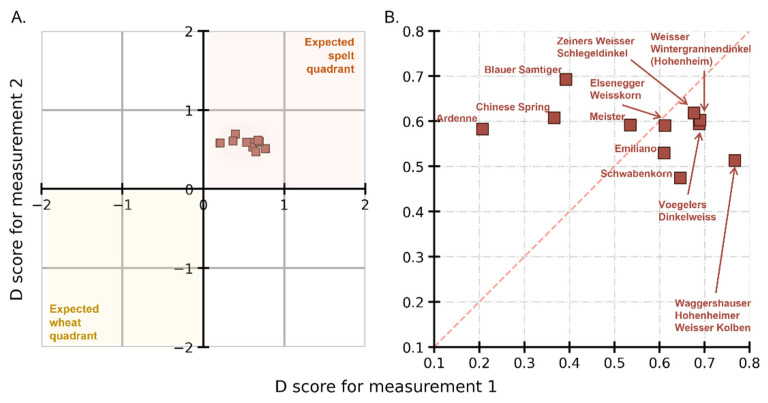
Youden plot showing the D scores for untypical spelt cultivars (**A**) with the magnified section in (**B**). Untypical spelt (brown squares) with their corresponding names, shown to be correctly predicted as spelt.

The next question was to check if other spelt cultivars (that were not used in the calibration set) could be correctly identified as spelt. Figure 4A shows the cluster of eleven cultivars (brown squares) lying in the spelt quadrant of the Youden plot, indicating correct classification. Figure 4B is the zoomed-in section of the plot showing the distribution of D scores with the corresponding cultivar name (see Supplementary Table S5 for a list of D scores for untypical spelt). The point cloud is in the first quadrant, showing the correct classification for spelt. The average D score is 0.57. Comparing this to the average of 1 for the spelt cultivars in the calibration set (Figure 2B), there is a difference in the prediction outcome of these untypical (for external validation) and typical (for the calibration set) spelt. This suggests that the fingerprints, as learned by the CNNs through the spectra of typical spelt, are dissimilar to those of untypical spelt. This could be linked to the evolving proteomic fingerprints of older cultivars of spelt (used in the calibration set) compared to the newer ones in untypical spelt. The larger spread of the points in the Youden plot for untypical spelt (Figure 4) in comparison to the spread of typical spelt (Figure 2) is a remarkable result. This can be owing to the dissimilarities between the learned and predicted fingerprints of typical and untypical spelt cultivars.

### 3.6. External Validation with Other Wheat Cultivars (Old Wheat Cultivars)

**Figure 5:**
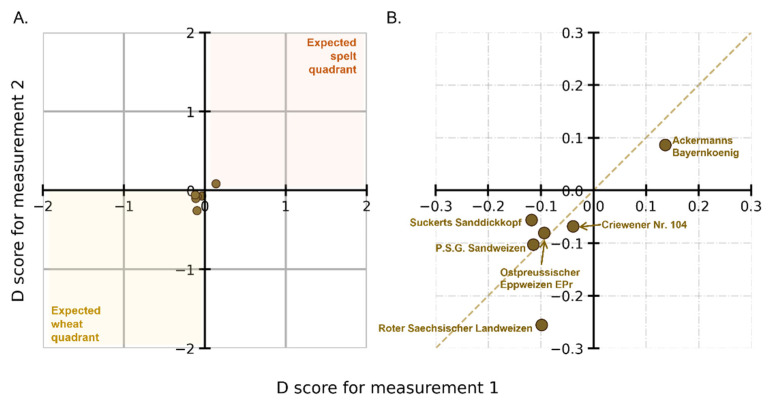
Youden plot showing the D scores for old wheat cultivars (**A**) with the magnified section in (**B**). The cultivars (brown circles) are shown to be not unambiguously classified as spelt or wheat.

On a similar line of inquiry, further investigations were made to determine whether old wheat cultivars, which were not part of the model building, can be distinguished from spelt (or wheat). Figure 5A shows the D scores for six cultivars with the zoomed view in Figure 5B (brown circles) (see Supplementary Table S6 for a list of D scores for old wheat). We see that even though five of the six cultivars lie in the wheat quadrant, i.e., D scores for five of the six cultivars are negative. However, for one cultivar, *Ackermanns Bayernkoenig*, it is positive. With zero as the decision threshold, it can be said that one cultivar is misclassified. However, all six cultivars are very close to the decision threshold. The mean D score for the other five is −0.1. Comparing this to the mean value of −1 for the wheat cultivars in the calibration set, there is a clear distancing from it.

By connecting these results to the pedigree of the cultivar, it may be possible to explain why they have either positive or close to zero D scores. For instance, *Ackermanns Bayernkoenig* an old cultivar, is a cross between wheat and spelt wheat, which could explain why CNN identifies it as being closer to spelt than wheat. Overall, these samples proved to be “challenging samples” for the method with the CNN models in their current form [31].

## 4. Discussion

This paper describes an NTM comprising LC-HRMS data acquisition as the wet lab component and using the 2-D MS1 spectral data as inputs for the CNN for classification tasks as the dry lab component. Note that the wet lab part involves duplicate measurements, which proves advantageous in capturing the variation due to sample preparation and measurement. In the dry lab part, the model development employs an NCV approach that relies on a calibration data set that is split into training and validation sets for each iteration. The study shows the merits of appropriately (and carefully) choosing datasets to train classification models. The classification probabilities obtained at the output layer of the CNN are transformed into a set of standardized numerical values that we call D scores. D scores provide a quantitative appraisal of the discrimination of two classes, and the results show how they also provide a visual representation of how clusters of samples are “related” to each other.

Catering to the question of differentiating spelt from wheat, the distribution of D scores shows that the CNN models are able to completely distinguish typical spelt and wheat cultivars with a very low risk of misclassification (<1%). The developed models were then put to the test to classify processed goods (spelt bread, spelt flour mix) and artificial mixes. These were correctly identified in all instances tested. We foresee the use of such an NTM on-site by laboratories of food production companies and official control, to aid with testing food authenticity and ensuring correct labeling of spelt products. After the labs have obtained the spectral measurement, it can be run through the models accessed by means of a suitable application interface, which will provide the D score. We believe this method adds to the battery of methods that have been reported thus far that utilize electrophoresis or molecular methods to distinguish spelt and wheat [12,15,19,21]. LC-HRMS measurements give a vast, high resolution, and high-fidelity database for the cost trade-off. However, when utilized appropriately by training CNNs using NCV, as described herein, it provides rapid, accurate and cost-effective results.

The CNN models developed as part of the dry lab procedure were further challenged with cultivars of spelt and wheat that were not part of the training. The untypical spelt were all correctly classified. D scores for old wheat cultivars were close to the decision, proving to be challenging samples for the NTM, with one out of six cultivars being misclassified. Systematic inclusion of such challenging cultivars along with additional ones that were not considered in the study would indeed help to upgrade the NTM. The discriminatory power of the method can be further improved by mobilizing the complete fragment-ion MS/MS spectrum.

An initial objective of the project was to make use of raw aggregated spectra without any alignment or peak picking, and this work describes a procedure to fulfill that objective. This is increasingly beneficial when (a) there is no a priori knowledge of which peaks to focus on, or (b) a combination pattern of several peaks is contributing to the identification or discrimination of the measured entity (in this case, spelt and wheat), or (c) processed food samples and matrix effects make it hard to detect the presence of specific marker peaks.

As previously discussed, bucketing of cultivars into spelt, wheat-spelt, and spelt-wheat are subjective with overlapping boundaries. All this leads to an unclear definition of spelt for both consumers and producers, which can be taken advantage of by the latter for economic benefits. Thus, raising questions about “what is true spelt?” As well as when does an untypical spelt cultivar stop being referred to as spelt? The NTM described here can help answer those questions by quantifying (using D scores) the deviations in characteristics (captured through the LC-MS fingerprint). The results described in Section 3.5 and Section 3.6 attest to the potential of the approaches described in this work to help get to a definition of spelt buckets. A further study involving the utilization of D scores to define what can be regarded as spelt (or not) is therefore proposed. For example, subjective buckets with diffused boundaries for spelt can be replaced by well-defined buckets by establishing suitable quantitative criteria (e.g., a D score greater than 0.5 results in true spelts).

A variety of NTMs involving proteomic- or metabolomic-based approaches are being developed to keep pace with new ways of deception with food substances. It is the view of the authors that NTMs in food testing clearly stand at a crossroads—with great promise for wide applicability and adoption that can be ushered in by establishing method validation schemes. Method validation schemes allow for the evaluation of the method’s performance, which can help standardize the method and bring it into routine use [31]. The provision of a complete method validation scheme is outside the scope of this work. However, a suitable scheme can be contemplated to utilize the quantitative D scores to evaluate the precision parameters. Consequently, performance characteristics like sensitivity and specificity, false-positive and false-negative rates can also be evaluated based on a chosen threshold score (D score of zero). The advantages of the proposed procedure of transforming the classification probabilities into standardized D scores become more evident when measurements across different laboratories can be directly compared in a validation study (single- or multi-laboratory).

From one perspective, the study is limited by the small dataset for training neural networks (calibration set). In such a scenario, one has to be careful with over-fitting issues. To alleviate these issues, the NCV approach was used, which helps achieve greater generalization on unseen data. This can be seen in the results for the external validation samples. Firstly, all processed goods were correctly classified. Secondly, untypical cultivars and old wheat cultivars were also meaningfully identified. The reader should bear in mind that this work does not aim and claim to provide the “best” models for classification of spelt vs. wheat with matchless classification metrics. Rather, the study aims to establish effective approaches and, thereby, contribute to the growing area of NTMs for food fraud.

In food fraud testing, one can imagine that data corresponding to “authentic” food samples will always be “limited,” as obtaining truly authentic samples might be burdensome or impractical. As in this study, knowledge about the real identity of the cultivar relies on elaborate biochemical tests and known cross-breeding histories. There is an increased role for the means by which the dataset is obtained or generated to reduce reliance on large datasets for model building. (a) Conducting duplicate measurements of cultivars, (b) selecting suitable cultivars as the two classes for the training, and (c) designing folds of the NCV approach are some of the procedures for systematic curation proposed in this work.

Overall, the described method can be easily (a) extended to include more cultivars and their mixes and (b) adapted for other application areas, such as the prediction of geographical identity. Furthermore, the modular nature of the method (wet lab + dry lab) means alternative approaches (e.g., different LC-MS instruments) can be used. The procedures, including duplicate measurements, NCV, and calculation of D scores, would still be applicable, as stated here.

## 5. Conclusions

This study describes a new NTM in which the wet lab component records the food fingerprint using LC-HRMS and the dry lab component utilizes CNN to identify the tested sample. The D score results show correct identification of relevant cultivars, with very low risk of misclassification. We see promise in the method’s usefulness not only in connection with the question of the authenticity of different food items and matrices but also, e.g., in characterizing blood plasma in connection with diagnostic, prognostic, and therapeutic research.

## Data Availability

Not applicable.

## Supplementary Materials

The following supporting information can be downloaded at: https://www.mdpi.com/article/10.3390/foods12010141/s1, Table S1: List of spelt and wheat varieties used in the study; Table S2A: Log odds and D scores for spelt cultivars in the calibration set; Table S2B: Log odds and D scores for wheat cultivars in the calibration set; Table S3: Summary of precision parameters for spelt and wheat; Table S4: Log odds and D scores for processed goods and artificial mixes; Table S5: Log odds and D scores for untypical spelt cultivars; Table S6: Log odds and D scores for old wheat cultivars.
