# Supplementary material for: Development of Non-Targeted Mass Spectrometry Method for Distinguishing Spelt and Wheat
Source: Foods. 2022 Dec 27;12(1):141. doi: 10.3390/foods12010141 (PMC9818861; doi:10.3390/foods12010141)
Supplement: Supplementary file 1 [file foods-12-00141-s001.zip › foods-207931-supplementary.pdf]

Development of Non-Targeted Mass Spectrometry Method for Distinguishing Spelt and Wheat

Kapil Nichani [1,2], Steffen Uhlig [3], Bertrand Colson [1], Karina Hettwer [1], Kirsten Simon [1], Josephine Bönick [4], Carsten Uhlig [5], Sabine Kemmlein [6], Manfred Stoyke [6,†], Petra Gowik [6], Gerd Huschek [7] and Harshadrai M. Rawel [2,*]

[1] QuoData GmbH, Prellerstr. 14, D-01309 Dresden, Germany
[2] Institute of Nutritional Science, University of Potsdam, Arthur-Scheunert-Allee 114-116, D-14558 Nuthetal, Germany
[3] QuoData GmbH, Fabeckstr. 43, D-14195 Berlin, Germany
[4] Bundesinstitut für Risikobewertung, Max-Dohrn-Str. 8-10, D-10589 Berlin, Germany
[5] Akees GmbH, Ansbacher Str. 11, D-10787 Berlin, Germany
[6] Bundesamt für Verbraucherschutz und Lebensmittelsicherheit, Diedersdorfer Weg. 1, D-12277 Berlin, Germany
[7] IGV-Institut für Getreideverarbeitung GmbH, Arthur-Scheunert-Allee 40/41, D-14558 Nuthetal, Germany
* Correspondence: rawel@uni-potsdam.de
† Currently independent researcher.

1. Supplementary Materials and Methods

1.1 List of spelt and wheat varieties

Table S1: List of spelt and wheat varieties used in the study

**Spelt cultivars in the calibration set**
Badekrone
Badensonne
Begrenzer Roter Spelz
Frankenkorn
Hohenloher
Holstenkorn
Lignee 24
Oberkulmer Rotkorn
Rottweiler Frühkorn
Schwabenspelz
Steiners Roter Tiroler
**Wheat cultivars in the calibration set**
Bernstein
Brilliant
Franz
JB Asano
Julius
Kerubino
Lear
Nordkap
Pionier
Produzent
RGT Reform

**Untypical spelt cultivars in external validation set**
Ardenne

Blauer Samtiger
Chinese Spring
Elsenegger Weisskorn
Meister
Waggershauser Hohenheimer Weisser Kolben
Zeiners Weisser Schlegeldinkel
Emiliano
Schwabenkorn
Voegelers Dinkel Weiss
Weisser Winter-Grannendinkel (Hohenheim)
**Old wheat cultivars in external validation set**
Criewener Nr 104
Ostpreussischer Eppweizen EPr
Roter Saechsischer Landweizen
Suckerts Sanddickkopf
Ackermanns Bayernkoenig
P.S.G. Sandweizen

2. Supplementary results

2.1 Internal validation: calibration set

The output of the convolutional neural networks (CNN) models are probability scores (p_i) which are transformed to log odds ($\ln(\frac{p_i}{1-p_i})$). The D scores are calculated using these log odds values. Both are listed below in Table S2 for the cultivars of the calibration set. An example calculation for the D score is described in the following.

The mean log odds are calculated for both the classes to be -7.66 and 7.303. The λ and θ values are then calculated according to Equations (S3) and (S4). The obtained λ and θ values are then used for the D score calculations for internal validation and external validation set.

$$\bar{\mu}_{spelt} = -7.660 \tag{S1}$$

$$\bar{\mu}_{wheat} = 7.303 \tag{S2}$$

$$\lambda = \frac{2}{\bar{\mu}_{spelt} - \bar{\mu}_{wheat}} = \frac{2}{-7.660 - 7.303} = -0.1336 \tag{S3}$$

$$\theta = 1 - \lambda \times \bar{\mu}_{spelt} = 1 - (-0.1336 \ \times -7.660) = -0.0238 \tag{S4}$$

For instance, the D score for measurement 1 of Badekrone spelt with log odds of -6.980 can be calculated as follows to give 0.909.

$$D_{Badekrone,M1} = (-6.980 \times -0.1336) - 0.0238 = 0.909 \tag{S5}$$

This way the D scores for all measurements are obtained.

Table S2A: Log odds and D scores for spelt cultivars in the calibration set.

**Spelt cultivars in the calibration set**	**Log odds**		**D score**	
	**Measurement 1**	**Measurement 2**	**Measurement 1**	**Measurement 2**
**Badekrone**	−6.980	−6.870	0.909	0.894
**Badensonne**	−4.758	−6.384	0.612	0.829
**Begrenzer Roter Spelz**	−8.958	−10.079	1.173	1.323
**Frankenkorn**	−7.178	−7.867	0.936	1.028
**Hohenloher**	−7.187	−7.715	0.937	1.007
**Holstenkorn**	−8.682	−8.476	1.137	1.109
**Lignee 24**	−7.403	−7.260	0.966	0.946
**Oberkulmer Rotkorn**	−8.877	−7.619	1.163	0.994
**Rottweiler Frühkorn**	−8.898	−7.029	1.165	0.916
**Schwabenspelz**	−8.409	−6.739	1.100	0.877
**Steiners Roter Tiroler**	−8.127	−7.029	1.062	0.916
**Measurement mean value**	−7.769	−7.551	1.015	0.985
**Class mean value**	−7.660		1	

Table S2B: Log odds and D scores for wheat cultivars in the calibration set

Wheat cultivars in the calibration set	Log odds		D score	
	Measurement 1	Measurement 2	Measurement 1	Measurement 2
**Bernstein**	7.229	7.745	−0.990	−1.059
**Brillant**	5.844	6.289	−0.805	−0.864
**Franz**	8.409	8.702	−1.148	−1.187
**JB Asano**	6.451	5.683	−0.886	−0.783
**Julius**	7.422	7.846	−1.016	−1.073
**Kerubino**	7.118	6.990	−0.975	−0.958
**Lear**	6.970	6.999	−0.955	−0.959
**Nordkap**	7.757	10.229	−1.061	−1.391
**Pionier**	7.044	6.852	−0.965	−0.940
**Produzent**	5.149	7.579	−0.712	−1.037
**RGT Reform**	8.101	8.264	−1.107	−1.128
**Measurement mean value**	7.045	7.562	−0.965	−1.035
**Class mean value**	7.303		−1	

2.2 Precision parameters

Table S3: Summary of precision parameters for spelt and wheat

	**Spelt**	**Wheat**
**Class mean D score value**	1	−1
**Intermediate SD**	0.075	0.074
**Classification SD**	0.393	0.391

2.2 External validation: processed goods and artificial mixes

Table S4 shows the average log odds and D scores obtained from predictions of the eleven NCV models, for the duplicate measurements of processed goods and artificial mix. The mean D score for artificial mixes is around 0.9.

Table S4: Log odds and D scores for processed goods and artificial mixes

**Processed goods and artificial mixes**	**Log odds**		**D score**	
	**Measurement 1**	**Measurement 2**	**Measurement 1**	**Measurement 2**
**Processed goods**				
Spelt flour mix	−6.133	−6.128	0.796	0.795
Spelt bread	−6.087	−5.830	0.790	0.755
Measurement mean value	−6.110	−5.979	0.793	0.775
Processed goods mean	−6.045		0.784	
**Artificial mixes**				
90% Oberkulmer Rotkorn +				
10% Bernstein	−7.437	−6.701	0.970	0.872
10% Brillant	−7.059	−6.298	0.920	0.818
10% Franz	−7.011	−6.201	0.913	0.805
10% JB Asano	−7.120	−6.589	0.928	0.857
10% Julius	−7.149	−6.595	0.932	0.858
10% Kerubino	−7.238	−6.619	0.944	0.861
10% Lear	−7.734	−6.991	1.010	0.911
10% Nordkap	−7.349	−6.372	0.958	0.828
10% Pionier	−7.199	−6.530	0.938	0.849
10% Produzent	−7.688	−6.817	1.004	0.887
10% RGT Reform	−7.043	−6.283	0.917	0.816
Measurement mean value	−7.275	−6.545	0.949	0.851
Artificial mix mean value	−6.910		0.9	

2.3 External validation: untypical spelt

Table S5 shows the average log odds and D scores obtained from predictions of the eleven NCV models, for the duplicate measurements of untypical spelt cultivars. The mean D score is 0.572.

Table S5: Log odds and D scores for untypical spelt cultivars

**Untypical spelt cultivars**	**Log odds**		**D score**	
	**Measurement 1**	**Measurement 2**	**Measurement 1**	**Measurement 2**
**Ardenne**	−1.726	−4.539	0.207	0.583
**Blauer Samtiger**	−3.114	−5.365	0.392	0.693
**Chinese Spring**	−2.919	−4.730	0.366	0.608
**Elsenegger Weisskorn**	−4.758	−4.596	0.612	0.590
**Emiliano**	−4.744	−4.142	0.610	0.530
**Meister**	−4.182	−4.604	0.535	0.592
**Schwabenkorn**	−5.012	−3.731	0.646	0.475
**Voegelers Dinkelweiss**	−5.325	−4.628	0.688	0.595
**Waggershauser Hohenheimer Weisser Kolben**	−5.919	−4.018	0.767	0.513
**Weißer Winter-Grannendinkel aus Hohenheim**	−5.341	−4.689	0.690	0.603
**Zeiners weisser Schlegeldinkel**	−5.240	−4.810	0.676	0.619
**Measurement mean value**	−4.389	−4.532	0.563	0.582
**Untypical spelt mean**	−4.460		0.572	

2.4 Extenal validation: old wheat

Table S6 shows the average log odds and D scores obtained from predictions of the eleven NCV models, for the duplicate measurements of old wheat cultivars. The mean D score for Ackermanns Bayernkönig is 0.11. And the mean D score for the duplicate measurements of Criewener Nr. 104, Ostpreußischer Eppweizen, P.S.G. Sandweizen, Roter Sächsischer Landweizen and Suckers Sanddickkopf is −0.1.

Table S6: Log odds and D scores for old wheat cultivars

Old wheat cultivars	Log odds		D score	
	Measurement 1	Measurement 2	Measurement 1	Measurement 2
**Ackermanns Bayernkönig**	−1.198	−0.824	0.136	0.086
**Criewener Nr. 104**	0.111	0.332	−0.039	−0.068
**Ostpreußischer Eppweizen**	0.521	0.424	−0.094	−0.081
**P.S.G. Sandweizen**	0.678	0.592	−0.114	−0.103
**Roter Sächsischer Land-weizen**	0.558	1.735	−0.098	−0.256
**Suckers Sanddickkopf**	0.704	0.240	−0.118	−0.056
**Measurement mean value**	0.229	0.417	−0.054	−0.080
**Old wheat mean value**	0.323		−0.067	
